# Sensory Evaluation of Effervescent Nutritional Supplements: Identification and Characterisation of Off-Tastes

**DOI:** 10.3390/molecules30040854

**Published:** 2025-02-13

**Authors:** Thomas Delompré, Christophe Martin, Loïc Briand, Christian Salles

**Affiliations:** 1 Centre des Sciences du Goût et de l’Alimentation, CNRS, INRAE, Institut Agro, Université Bourgogne Europe, F-21000 Dijon, France; thomas.delompre@inrae.fr (T.D.); christophe.martin@inrae.fr (C.M.); loic.briand@inrae.fr (L.B.); 2 CNRS, INRAE, PROBE Research Infrastructure, ChemoSens Facility, F-21000 Dijon, France

**Keywords:** sensory analysis, nutritional supplement, perception, flavour, cross-modal perceptual interaction

## Abstract

Nutritional supplements are often characterised by unpleasant tastes or aftertastes, primarily due to the presence of vitamins, minerals, and amino acids as active compounds. These taste defects can be masked by sweeteners or specific flavourings. However, the development of such strategies requires a thorough understanding of the sensory characteristics of nutritional supplements. In the present study, the sensory properties of four effervescent nutritional supplements, differing in composition, were evaluated using Quantitative Descriptive Analysis (QDA) across three modalities: orthonasal and retronasal odour perception, as well as aftertaste and aroma persistence. Bitterness, astringency, and metallic sensations were found to be responsible for the negative sensory attributes of the products in solution. The addition of flavouring agents was found to have either a positive or negative effect on the taste characteristics of the supplements. Indeed, certain fruity notes enhanced sweet and sour sensations and were found to mask negative sensory perceptions, although this effect varied depending both on the nature of the nutritional supplement and on the QDA modalities, mainly due to the oral process progressing. A better understanding of these perceptual interactions could provide a solution for masking strategies, potentially reducing the use of additives that can be expensive and detrimental to health.

## 1. Introduction

More and more people across the world use nutritional supplements, in particular to prevent or treat certain daily inconveniences such as fatigue, a lack of concentration, vitamin/mineral deficiencies, minor gastroenterological and dermatological disorders, and vaginal irritations. Oral nutritional supplements are also prescribed to supply nutrients to those with deficiencies which can be due to certain pathologies and to restore the proper development and functions of the body [1,2]. Manufacturers are continuously developing new formulas that differ in their flavouring, content, and galenic form to meet new market demands and economic and/or regulatory constraints, and to comply with specific populations. Efforts are now focused on the improvement of specificity and efficiency, in particular in formulation, to assure better release control when necessary. However, nutritional supplements are often characterised by taste defects: bitterness, astringency, and a metallic sensation, constituting an important technological barrier to consumer acceptance. This can be explained by the presence of nutrients such as vitamins, minerals, and amino acids known to be at the origin of these negative perceptual sensations [3]. As these components are also the active compounds which are in direct contact with the taste detection system in the mouth when the supplement is ingested, a strategy based on substitution to mask these taste defects is excluded. Therefore, effective masking strategies targeting the lowering of taste defects both at the peripheral and central level appear to be the most promising. However, little data are available concerning the quantitative sensory characteristics of nutritional supplements. Temporal dominance of sensations (TDS) and temporal check-all-that-apply (TCATA) methods were chosen to characterise temporal sensory perceptions during the consumption of orally disintegrating tablets varying in galenic forms and flavouring [4]. Minerals, amino acids, and vitamins with bitter properties were found to be able to activate one or more of the 25 bitter taste receptors (TAS2Rs) involved in bitter taste detection [5,6,7]. In particular, the human bitter taste detection threshold for four vitamins in aqueous solution—vitamin B1 (thiamine hydrochloride), vitamin B2 (riboflavin phosphate), vitamin B3 (niacinamide), and vitamin B6 (pyridoxine hydrochloride)—which exhibit a strong bitter taste was compared to their half maximal effective concentration (EC50) and their cellular bitter taste threshold [8]. The results suggest that their associated bitter taste perception can be explained by interactions with TAS2Rs.

However, qualitative data alone do not make it possible to determine the real contribution of these perceptions in the genesis of bad tastes. Thus, an accurate quantitative sensory description of nutritional supplements, in particular for taste and aroma characteristics, is necessary before proceeding further in the elaboration of masking strategies based on flavour perception. Indeed, the improving of the formulation of supplements by flavouring could make them more acceptable to users, in particular in masking off-flavours through cross-modal perceptual interactions [9]. In this study, we focused mainly on a particular galenic form that has been present on the nutraceuticals market for many years: effervescent nutritional supplements. Their low representation in the current market for nutritional supplements (4.5% compared with 24.5% for ‘gummy’ forms, for example) means that they are now one of the market players that is often overlooked. However, their many advantages [10], such as ease of ingestion, rapid and enhanced absorption of the active compounds they contain, and perfect compatibility with stomach pH, make them very useful in nutritional supplements for people suffering from swallowing disorders or certain oral and digestive cancers.

To date, although a few sensory analyses have been carried out on various nutritional supplements (protein-supplemented liquid and orodispersible galenic forms) [11], no data on the organoleptic qualities of effervescent nutritional supplements enriched with vitamins and minerals are available in the scientific literature. However, it is frequently observed that taste and flavouring play a decisive role in the reluctance to consume them. The objectives of this work were to accurately determine the nature of these negative perceptual sensations, to quantify them, and to specify their origin with the aim of improving their organoleptic quality.

## 2. Results

### 2.1. The Sensory Characterisation of the Nutritional Supplements

#### 2.1.1. Orthonasal Olfactory Properties (Smell)

The results of the evaluation of sensory characteristics by orthonasal airway of the four nutritional supplements are presented in Table 1. It is noteworthy that the four nutritional supplements, CN1, CN2, CN3, and CN4, are characterised by very distinct olfactory sensory attributes (smell).

Among all the descriptors evaluated, only the sensory attribute “chemical orange” is a sensory characteristic applicable to CN1. CN2 is significantly characterised by olfactory notes of “passion”, “exotic fruits”, “yellow fruits”, and “red fruits”. Citrus olfactory notes evoking a sensation of freshness such as “fresh orange” and “mandarin” are sensory characteristics specific to CN3. Finally, CN4 is characterised by “mature orange” and “passion” olfactory notes.

#### 2.1.2. Retronasal Olfaction

During this tasting phase, which therefore corresponds to the ingestion of the entire product, the panellists were asked to evaluate the intensity of sensory attributes (Table 2).

**Figure 1:**
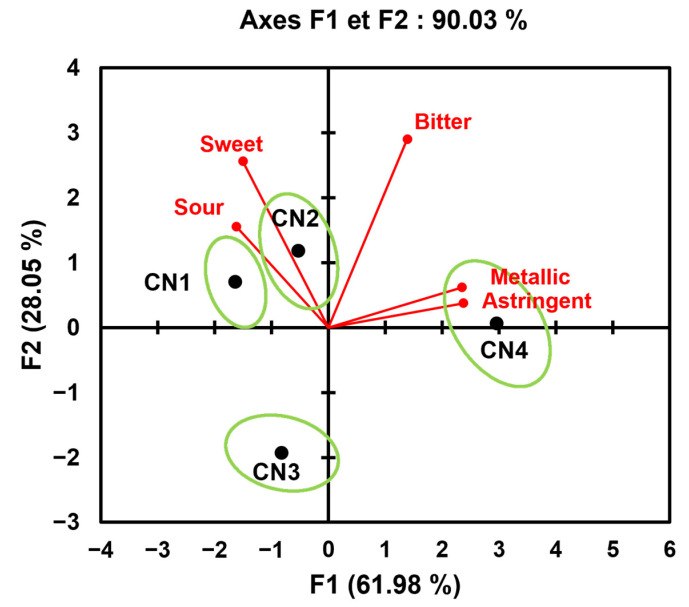
Principal component analysis in a correlation matrix for the taste, astringent and metallic attributes of the nutritional supplements (CN1–4) in the “during tasting” segment (biplot representation).

The taste, astringent, and metallic perceptions are presented in Figure 1. The F1 axis is strongly correlated with the sensory attributes “metallic” and “astringent” in its positive section and “sourness” in its negative section. The F2 axis is represented by the sensory attributes “sweet” and “bitter”. The F1 axis therefore separates the products according to their sourness (CN1 and CN2) and according to the taste attributes “astringency” and “metallic” (CN4). The F2 axis makes it possible to differentiate nutritional supplements based on perceived bitterness and sweetness. CN2 therefore has a more pronounced bitter taste than CN1 but a bitter taste equivalent to CN4, as well as higher sweetness. CN3 is only characterised by the absence of bitterness, without any of its own taste characteristics.

**Figure 2:**
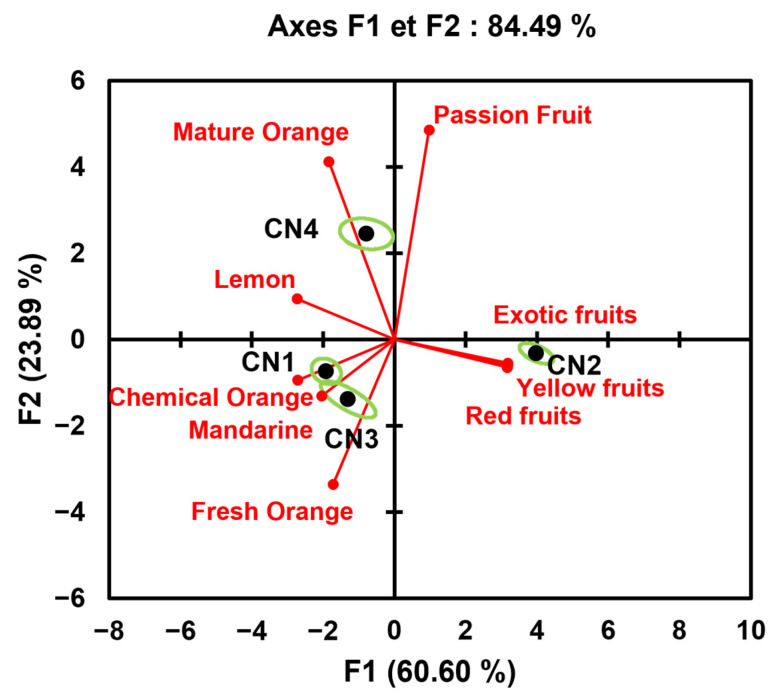
Principal component analysis in a correlation matrix for the retronasal odour attributes of the nutritional supplements (CN1–4) in the “during tasting” segment (biplot representation).

The retronasal aroma perceptions are presented in Figure 2. The F1 axis makes it possible to differentiate nutritional supplements according to the attributes “red fruits”, “exotic fruits” and yellow fruits” in its positive section and the attributes “chemical orange” and “tangerine” in its negative section. The F2 axis is strongly correlated with mature orange, passion fruit and fresh orange notes. CN1 and CN3 have common sensory characteristics, namely, “chemical orange” and “mandarin” attributes, but are differentiated by the “fresh orange” attribute, which is significantly more marked in CN3. CN2 and CN4 are, respectively, characterised by the attributes “red fruits”, “exotic fruits” and “yellow fruits” and “mature orange” and “passion fruit”.

The results of the two-way ANOVA (model: subject + product + subject × product) presented in Table 2 show significant differences between the products. For example, the average intensity of the sensory attribute “mature orange” is significantly different for each nutritional supplement.

#### 2.1.3. Aftertaste and Retronasal Odour Persistence

This perception corresponds to the sensations perceived after swallowing the nutritional supplement solutions. These late sensory perceptions are commonly called “aftertaste” for taste attributes and “retronasal odour persistence” for aroma attributes.

The average intensity values of each of the aftertastes evaluated for each nutritional supplement are reported in Table 3. The nutritional supplements have aftertastes of variable intensity, whether they are common or not, with positive valence and/or negative valence. All products are characterised by sour and sweet aftertastes, although significant differences are observable. CN1 and CN4 are, respectively, characterised by “salty” and “metallic” and “astringent” aftertastes. The “bitter” aftertaste is a sensory attribute encountered in CN1, CN2 and CN4 with an intensity varying between 1.2 and 1.7. The relatively low average intensity of this attribute in CN3 seems to be the one and only sensory characteristic specific to it.

### 2.2. Effect of Retronasal Odour Perception on Taste Perceptions

A sensory evaluation of the four nutritional supplements was also carried out in a blocked airway condition in order to eliminate the potential effect of retronasal odour perceptions on taste perceptions, during and/or after tasting the product.

For the four nutritional supplements, significant differences are observable for at least three sensory taste attributes during the two evaluation segments (during and after tasting).

The data are summarised in Table 4. For CN1, CN2 and CN3, aromatisation leads to a significant increase in the average intensity of the “sweet” attribute in the two evaluation phases, a descriptor with strong positive hedonic valence strongly correlated with the notion of pleasure. Conversely, the average intensity of unattractive attributes such as “bitter”, “astringent” or “metallic” decreases significantly (depending on the nutritional supplement and the evaluation segment considered). The exception is CN4, which follows the opposite logic to that stated previously, since its flavouring is the cause of a significant increase in the “bitter” attribute during tasting.

We can see that odourants evoking notes of citrus or exotic fruits reinforce the perception of the “sour” aftertaste. The “metallic” attribute, whose average intensity is significantly higher “during tasting” and “after tasting” for CN4, completely disappears during sensory evaluation in the blocked airway condition. Although suitable flavouring can significantly reduce the perceptions of certain attributes, it cannot alone be responsible for the total disappearance of the sensory characteristics of a product. This phenomenon is linked to the volatile properties of the molecules responsible for this sensory quality, which, like aromatic molecules, will be perceived by the olfactory organ via the retronasal route [12,13]. The flavouring of different nutritional supplements does not seem to significantly affect perceptions of the “salty” attribute, except for CN1.

## 3. Discussion

### 3.1. The Relationship Between the Composition of Nutritional Supplements and Their Sensory Characteristics

Taste sensory perceptions are linked to the composition of these nutritional supplements. Generally speaking, the nutritional supplements studied are “sour” and “sweet”, during tasting and a few seconds after complete ingestion. The observed sourness may be related to the presence of acid compounds, such as malic acid and citric acid, which are present in the excipient composition of each nutritional supplement. Indeed, when dissolved in water for consumption, their respective concentration is higher than their threshold perception value, estimated at around 5 × 10^−^^6^ M. The relative concentration of one compared to the other does not seem to affect either the perception of sourness or that of astringency [14].

We also note the presence of ascorbic acid (vitamin C) as an active compound in the four formulations at rather high concentrations, between 60 and 1000 mg per tablet (Table 1), the impact of which on the sour perception component of food supplements has already been reported [3]. With the threshold perception value of ascorbic acid being estimated in the range of 0.28–0.75 mM [15], its concentration in the four CN solutions indicates that it is certainly also active in sourness perception. Although to our knowledge, no perceptual intramodal interaction between these acids was reported, they probably additionally act in the overall sour perception of these nutritional supplements. Moreover, even if citric and malic acids are parts of the excipient fraction of the nutritional supplements, they were found to have a positive effect on intragastric urease activity, while that of ascorbic acid is very limited [16].

Regarding the sweet perception of these nutritional supplements, its pronounced intensity is linked to the presence of “intense” sweeteners and “bulking” agents whose objectives are numerous, with a sweetening power of between 0.5 and 0.9. They are incorporated in large quantities, representing up to 80% of the final mass of the tablet. Although their sweetening power is relatively low, the large quantities used give them a significant sweetening capacity.

The sensory attribute “bitter” was evaluated and found to have an average intensity greater than 1 for the majority of products (CN1, CN2 and CN4). This pronounced and persistent bitterness can be explained by the incorporation of active molecules, in native or analogous form, such as vitamins, minerals or plant extracts. The majority of these active compounds are not found in CN3, which differs from the other three nutritional supplements in its low bitterness. Some water-soluble vitamin analogues belonging to the B vitamin family such as thiamine hydrochloride, riboflavin phosphate, nicotinamide or pyridoxine hydrochloride are bitter [3]. The incorporation of magnesium sulphate into CN1 and CN4 could contribute to reinforcing the bitterness of these products [5,17], just like the manganese sulphate present in CN4.

Some mineral salts used in these nutritional preparations also have other negative sensory qualities. They are responsible, among other things, for the more marked “metallic” and “astringent” perceptions when CN4 is consumed [18,19].

CO2 (and its dissociation metabolites such as carbonic acid) has been identified as responsible for increasing the bitter flavour of carbonated products such as beer [20,21], a bitterness observed in the four nutritional supplements. Furthermore, by contributing to the lowering of pH in the oral cavity and the remodelling of interactions between salivary proteins, effervescence is linked to the increase in the sensation of astringency [22], which is found in all of the products studied. Studies demonstrate the effects of effervescence on sweet and sour perceptions, which depend on the average values of their initial intensity within the product evaluated. In low concentrations, effervescence reinforces the sour perception of citric or phosphoric acid solutions, while it helps to mask the sweet perceptions of sucrose or aspartame solutions [22,23,24].

We also observed changes in relative intensity for the perception of certain notes depending on the nutritional supplement during the three oropharyngeal phases, once the supplement is completely dissolved in water. This is the case for the chemical orange attribute, which, in comparison to CN1, becomes more intense after being put into the mouth than when experienced by simple olfaction. This observation could be explained by a difference in the physical process in the mouth, such as a greater retention of the aroma compounds responsible for this note in the mouth, by the matrix or the oral mucosa. However, we can also notice that during consumption, CN3 and CN4 are less sour and less sweet than CN1, taste attributes which could mask this chemical orange note through cross-modal perceptual interactions.

It would therefore be valuable to complement these quantitative sensory analyses with consumer tests and overall flavour appreciation ratings and/or ratings specific to a particular taste. The data obtained could, through statistical correlation with quantitative data, help identify the sensory perceptions that lead to consumer acceptance or rejection [25]. A sensory approach combining conventional profiling with sequential appreciation ratings, aligned with an ingestion process involving repeated swallowing, also appears to be an appropriate methodology for evaluating products such as effervescent nutritional supplements, known for their persistent negative sensory perceptions [26].

### 3.2. Perceptual Interactions

These nutritional supplements, when dissolved in water, constitute a complex mixture of compounds of different perception qualities which can interact with each other either at the peripheral level or at the central level. Citric and malic acids are the major organic acids in fruits and are responsible for their sourness [14,27,28], and are highly present in each of the four CN supplements.

The comparison of taste, metallic and astringent intensities by blocking or not blocking the perception of aromas shows the numerous effects of retronasal odour perception on these attributes, sometimes different from one product to another, but going in either direction with one masking the other in the sense of reinforcing taste, astringency and metallic perceptions. However, since retronasal odour perception acts on several descriptors at the same time, analysis is complex, because we cannot exclude indirect effects due to variations in the intensity of another attribute. For example, for sourness, only CN4 shows an effect of masking this taste through retronasal odour perception. However, we also observed that bitterness intensity increases with retronasal odour perception. This masking of sourness could also be explained by the effect of a more intense perception of bitterness [29].

Concerning sweetness, the reinforcement of this perception by retronasal odour perception is explained by cross-modal perceptual interactions with the fruity notes associated with sweetness, as has already been described [9]. This effect is not observed for CN4, although fruity notes are perceived. This absence can be explained by the presence of a more intense metallic note in this product which could either mask or deteriorate the overall fruity note in such a way that it is no longer recognisable as such to the panellists. For CN4, the increase in bitterness through the perception of aromas could be a consequence of the increase in the intensity of metallic perception, which is now treated as aroma perception [13,30,31]. Overall, we observed a masking effect of retronasal odour perception on unfavourable perceptions such as bitterness and astringency for the four products, which is relevant for developing a strategy for masking these unfavourable perceptions in this type of product. However, at the same time, we observed an increase in the intensity of a metallic note, which is also unfavourable. One explanation could be a masking effect of bitterness and astringency on this perception. When the intensity of these perceptions is reduced by the perception of aromas, the metallic note would then be revealed.

Concerning the “after tasting” segment, we observed, to a lesser extent, the effects of the reinforcement and masking of sensory attributes by the persistent retronasal odour perception, but, in most cases, in a different manner compared to what was observed in the during tasting segment. This can be explained by the effects of rinsing with saliva and the progressive exhaustion of the ingested material leading to changes in the balance and ratio between the different stimuli released from the product, depending on their more or less significant persistence in the oropharyngeal cavity. Furthermore, we consistently observed a strengthening of the sweet flavour through retronasal odour perception for all products when they were tasted without blocking the nasal airway. However, for CN2 and CN3, the strengthening of the sour perception despite the strengthening of the intensity of the sweet perception, which usually exerts a masking effect on sourness, could be explained at least partially by a reduction in bitterness [29].

### 3.3. The Outcomes of the Study

These findings highlight the importance of considering all available sensory information about active compounds and other formulation agents used in the development of nutritional supplements, even before the formulation process begins. This study demonstrates, through a comparison of the sensory profiles of four effervescent nutritional supplements, that the unique sensory properties of each compound significantly influence the organoleptic quality of the final product. Although no other studies have specifically addressed effervescent products enriched with vitamins and minerals, similar results have been observed in various food matrices [32]. Previous research has also shown that omission tests are an effective strategy to determine the precise contribution of a compound to a product’s sensory characteristics [33]. Applying this methodology to effervescent nutritional supplements—by omitting a compound or group of compounds from the formulation and evaluating the resulting product with an expert panel—could yield valuable insights. One potential avenue for improving the organoleptic qualities of effervescent supplements may be to incorporate analogous compounds (vitamins, minerals and formulation agents) with neutral sensory properties to minimise their impact on the product’s overall organoleptic profile.

Our study also reveals, for the first time, the significance of interaction phenomena between taste-active and aroma-active molecules within effervescent nutritional supplements. We demonstrated that flavouring significantly affects the taste qualities of the studied products. These interaction phenomena, commonly referred to as “odour-induced taste enhancement”, have been observed in many processed foods and experimentally reconstructed matrices. For instance, it has been shown that vanilla or strawberry flavouring enhances the perception of sweetness [34]. A previous laboratory study highlighted a reduction in the bitterness perception of orodispersible nutritional supplements following the addition of a red fruit flavour [4]. Although the mechanisms underlying these interaction phenomena remain poorly understood, this represents a promising approach for improving the organoleptic qualities of such products. Further research on new aromatic combinations and their impact on sensory perceptions would be valuable.

Numerous studies have also reported other types of interactions within complex food matrices. While this study did not explore these interactions in detail, they remain a potential avenue for improving the organoleptic quality of these products. Already utilised in the formulation of various food matrices, such interactions include those between taste-active molecules. For example, it has been demonstrated that a suprathreshold mixture of sucrose and quinine exhibits a sweetness intensity lower than that of an equimolar sucrose solution and a bitterness intensity lower than that of an equimolar quinine solution [35]. These interactions thus modulate the intensity of both mixed flavours in varying proportions, depending on the stimuli, through enhancing or masking effects [29].

## 4. Materials and Methods

### 4.1. Experimental Conditions

The room in which the sensory experiments took place met the basic requirements dictated by the AFNOR V 09-105 standard [36]: a uniformly light, ventilated room, with stable temperature and humidity (20 °C), in a quiet space and in separate booths. Data acquisition was carried out using TimeSens sensory analysis software (TimeSens version 2.00, Dijon, France). During the sensory profiling sessions, the samples were served at 20 °C (room temperature) in opaque 100 mL cups labelled with three-digit codes. After each tasting, participants were instructed to take a one-minute break and cleanse their mouth with water and rusks.

### 4.2. Products

Four effervescent nutritional supplements named CN1, CN2, CN3 and CN4 provided by BAYER SAS (Global innovation Centre, Gaillard, France) were evaluated. Table 5 summarises their composition and flavouring. As excipients, each contained malic and citric acids, sodium carbonate, sucrose, polyols (mannitol, sorbitol, isomalt, maltitol), sweeteners (aspartame, acesulfame potassium, sucralose), antifoaming agents (polysorbate 80, polysorbate 60, sucrose esters) and disintegrants (crospovidone, croscarmellose sodium).

The tablets were stored in a room provided for this purpose (temperature- and humidity-controlled).

### 4.3. Sensory Profile Procedure

#### 4.3.1. The Recruitment and Selection of the Panel

An ethical committee approved this study (Personal Protection Committee of Rennes—Ouest V, protocol code 2018A01342-53), and all panellists signed an informed consent form. Participants suffering from food allergies or cardiovascular, kidney and/or liver problems, as well as pregnant women, were excluded from the study. A total of 23 people (21 women and 2 men, 22–56 years old) were recruited for the training and sensory analysis sessions (internal panel). During two one-hour sessions, the panellists were assessed on their sensory acuity in order to determine possible disorders of taste and/or olfactory perception (taste and odour sensitivity and recognition tests) [37]. A meeting was also organised to inform future panellists of the conditions required to participate in this panel (interest in the study, motivation, punctuality, objectivity). After an interpretation of the tests, the 23 candidates met the conditions set and thus constituted the panel of expert tasters.

#### 4.3.2. Presentation of Sensory Analysis to Panellists

Three different phases were considered for sensory evaluation: (i) orthonasal olfactory properties (smell—before tasting), (ii) sensory properties experienced with the product in the mouth (retronasal olfactory properties, taste and trigeminal sensations—during tasting), and (iii) aftertastes after spitting out the sample (residual retronasal olfactory properties, tastes and trigeminal sensations—after tasting). During a one-hour roundtable, the panellists were introduced to the notion of taste and aroma, collectively referred to as “flavour”, and aftertaste. The sensory systems associated with these perceptions were also presented. In order to study the possible gustatory impact of flavouring agents, the evaluations of the “during tasting” and “after tasting” segments were carried out by blocking or not blocking the air flow in the retronasal route (condition “with nose-clip” and “without nose-clip”).

#### 4.3.3. Establishment of Descriptor List

The panellists were instructed to refrain from drinking, eating or wearing perfume for one hour prior to the sensory analysis. The four primary tastes (sour, sweet, salty, and bitter) as well as astringency and metallic sensation were automatically included in the list of taste descriptors and trigeminal sensations. The list of olfactory descriptors characteristic of each of the products was produced in accordance with the method described by Moskowitz [38] during two sessions of one hour each. During each of these two sessions, the panellists tasted five products representative of the diversity of products evaluated and generated as many odour (orthonasal olfaction) and aroma (retronasal olfaction) descriptors as they could. During two additional sessions, the panellists focused on identifying descriptors that could discriminate between the samples and defined each taste, odour and aroma descriptor using a standard and a verbal definition. By the end of this phase, a final list, composed of 15 descriptors (9 olfactory/aromatic attributes and 6 taste/trigeminal attributes), was retained for the sensory profiles of the products. The sensory attributes, their definitions and their reference samples as well as their assessment procedures are presented in Table 6 and Table 7.

#### 4.3.4. Training of Sensory Panel and Monitoring of Individual Performances

The sensory panel was trained in accordance with AFNOR-ISO 8586 standard [39]. The sensory performance of the panellists was optimised through long-term continuous training for 4 months, with one session per week of a 1 h duration. Before a consensus on the rating of the different descriptors could be obtained, it was necessary to ensure that they were memorised by future sensory experts. Odour and taste recognition tests included in the list of descriptors were therefore carried out. The tests consisted of associating representative samples of a sensation, presented randomly, with the corresponding term in the list of descriptors. Once the sensory descriptors had been memorised and recognised, learning focused on rating their intensity. First, ranking tests were carried out to familiarise the panellists with the intensity evaluation task. The objective was to classify four to five samples (randomly coded) in increasing or decreasing order of intensity. Subsequently, the judges were asked to assign an intensity score to these samples using a structured scale of 10 cm (1 cm representing 1 point) limited at each end by reference samples for 0 (zero intensity) and 10 (strong intensity).

All of these tests were conducted with and without the nose-clip, in order to familiarise the panellists with the use of this device, designed to block the retronasal perception of volatile compounds. At the end of these different training stages, the performance of the panellists was evaluated by carrying out an analysis of the sensory profile of products similar to the nutritional supplements studied. This step aimed to check the discriminatory power of the sensory panel and its repeatability but also the agreement between the different evaluators who compose it. The sensory profile of three products was determined in triplicate and under real sensory evaluation conditions. The data were collected by software dedicated to sensory analysis (TimeSens version 2.00, Dijon, France) and evaluated using the sensory analysis module available in XLSTAT-Sensory statistical software (version 2020.2.3, Addinsoft, Paris, France). The training data were analysed using ANOVA, with the following model: subject + product + session + subject × product + error (random effects of subject and session). The results of this analysis showed that the performance of 16 out of the 23 panellists was good in terms of discriminative power and repeatability, and that the agreement among these 16 judges was also good. These 16 panellists were selected to participate in the measurement sessions, while the 7 panellists with lower performance did not take part in these sessions [40].

#### 4.3.5. Measurement Sessions

The sensory evaluation of the four nutritional supplements (CN1, CN2, CN3 and CN4) was conducted with the 16 judges selected after training (15 women and 1 man). The measurements were repeated two times for each product tested and for each evaluation condition (with and without a nose-clip). To clarify, the evaluation with a nose-clip, which eliminates the retronasal perception of volatile compounds, aimed to assess the potential gustatory impact of the flavouring agents.

The samples evaluated consisted of a quarter of an effervescent tablet diluted in 50 mL of water, in accordance with the supplier’s recommendations. Evian mineral water (Evian, France) was used for the preparations. All samples were presented in white cups, randomly coded with a three-digit number.

For each measurement session, sample evaluation was performed monadically, and the order of sample presentation followed a Williams Latin Square design to balance order and carryover effects (a different Latin square was used for each session). For the evaluation without a nose-clip, the sensory attributes were assessed in the following order: first, orthonasal olfactory properties (smell), followed by the sensory properties experienced with the product in the mouth (retronasal olfactory properties, taste and trigeminal sensations), and finally, aftertastes after spitting out the sample (residual retronasal olfactory properties, tastes and trigeminal sensations).

For the evaluation with a nose-clip, the order of evaluation was as follows: sensory properties with the product in the mouth (flavour and trigeminal sensations), followed by aftertastes (residual flavour and trigeminal sensations).

### 4.4. Statistical Analyses

The statistical tests used to process the data from the sensory analysis of the four nutritional supplements were selected in accordance with the available scientific literature. Analysis of variance (ANOVA), Tukey’s Honest Significant Difference (HSD) multiple comparison test (*p* < 0.05), and principal component analysis (PCA) were carried out using XLSTAT-sensory software (version 2020.2.3, Addinsoft, Paris, France).

For each sensory descriptor, an ANOVA was conducted with the product and panellist as the main effects, along with their interaction, to evaluate panel performance and identify which descriptors significantly differentiate the products [41,42]. The model applied was as follows: descriptor intensity = panellist + product + panellist × product + error (random panellist effect). For each descriptor, the panel’s ability to differentiate between products was evaluated using the F-value for the product effect (the panel was discriminating if the product effect was significant). These results are presented in the Supplementary Materials. For each descriptor, post hoc multiple pairwise comparisons (Tukey’s HSD, with a significance level set at 5%) were performed to compare the products pairwise.

A normalised principal component analysis (PCA, biplot representation) of matrix correlation with all sensory descriptors was carried out in order to visualise, on the same, single two-dimensional plane, the correlation between the sensory attributes and the nutritional supplements evaluated. To enhance the readability of PCA, only significantly different means are presented.

Moreover, an additional ANOVA and Tukey’s HSD tests (*p* < 0.05%) were performed to determine the impact of flavouring on taste perceptions for each nutritional supplement. The model applied was as follows: subject + product + condition + condition × product + error. These analyses were conducted using the sensory profiling data averaged across the two repetitions for each panellist, each product and each condition.

## 5. Conclusions

This study aimed to establish the sensory profile of effervescent nutritional supplements dissolved in water by differentiating the three oropharyngeal phases: before ingestion (orthonasal olfactory properties), during ingestion—sensory properties experienced with the product in the mouth (retronasal olfactory properties, taste and trigeminal sensations)—and aftertastes—after spitting out the sample (residual aromas, flavours and trigeminal sensations). We observed that the nutritional supplements were characterised by very distinct olfactory sensory attributes, regardless of the perception modalities. During ingestion, each nutritional supplement was differentiated from the others by one or more attributes: sweet, bitter, sour, astringent and metallic. Similarly, differences between products were found in the retronasal perception of odorous attributes. Sour and sweet aftertastes were observed for each product, while the intensity of other attributes—salty, metallic, astringent and bitter—allowed for further differentiation. Moreover, retronasal odour perception, due to the flavouring of the products, was found to positively or negatively influence other perceptions, such as taste, metallic and astringent sensations, during both ingestion and the aftertaste phase. Hypotheses regarding the mechanisms underlying these sensory modifications have been proposed.

Based on these findings, the main perspectives are as follows: (i) understanding these interactions between taste and retronasal odour perceptions could help optimise strategies for masking undesirable tastes, potentially avoiding the use of sweeteners or costly encapsulation techniques, and (ii) reducing the incorporation of substances such as salts and sugars in line with nutritional recommendations.

## Figures and Tables

**Table 1:** The average intensity (±standard error) of each odorant sensory attribute in the “before tasting” segment for the nutritional supplements.

Attribute	CN1	CN2	CN3	CN4
Orthonasal odour				
Chemical orange	5.60 ± 0.078 ^a^	0.56 ± 0.078 ^d^	3.46 ± 0.078 ^b^	2.40 ± 0.078 ^c^
Fresh orange	1.10 ± 0.070 ^b^	0.35 ± 0.070 ^c^	3.13 ± 0.070 ^a^	0.43 ± 0.070 ^c^
Mature orange	1.29 ± 0.073 ^b^	0.42 ± 0.073 ^c^	0.35 ± 0.073 ^c^	2.65 ± 0.073 ^a^
Lemon	0.16 ± 0.050 ^a^	0.22 ± 0.050 ^a^	0.33 ± 0.050 ^a^	0.25 ± 0.050 ^a^
Mandarin	0.37 ± 0.066 ^b^	0.18 ± 0.066 ^b^	1.21 ± 0.066 ^a^	0.33 ± 0.066 ^b^
Passion fruit	0.33 ± 0.059 ^c^	2.19 ± 0.059 ^b^	0.29 ± 0.059 ^c^	3.90 ± 0.059 ^a^
Exotic fruits	0.18 ± 0.064 ^c^	2.34 ± 0.064 ^a^	0.29 ± 0.064 ^c^	0.55 ± 0.064 ^b^
Yellow fruits	0.19 ± 0.112 ^b^	1.39 ± 0.112 ^a^	0.14 ± 0.112 ^b^	0.36 ± 0.112 ^b^
Red fruits	0.11 ± 0.039 ^b^	0.13 ± 0.039 ^a^	0.21 ± 0.039 ^b^	0.15 ± 0.039 ^b^

The estimated means in the same line identified by different letters are considered significantly different (Tukey test, *p* < 0.05).

**Table 2:** Average intensity (±standard error) of each aromatic and taste sensory attribute in the “during tasting” segment for the nutritional supplements.

Attributes	CN1	CN2	CN3	CN4
Retronasal odour				
Chemical orange	5.69 ± 0.097 ^a^	0.55 ± 0.097 ^c^	2.88 ± 0.097 ^b^	2.60 ± 0.097 ^b^
Fresh orange	1.15 ± 0.060 ^b^	0.26 ± 0.060 ^c^	2.83 ± 0.060 ^a^	0.37 ± 0.060 ^c^
Mature orange	1.48 ± 0.041 ^b^	0.25 ± 0.041 ^d^	0.62 ± 0.041 ^c^	2.49 ± 0.041 ^a^
Lemon	0.35 ± 0.050 ^ab^	0.18 ± 0.050 ^b^	0.47 ± 0.050 ^a^	0.44 ± 0.050 ^a^
Mandarin	1.05 ± 0.070 ^a^	0.21 ± 0.070 ^b^	0.37 ± 0.070 ^b^	0.35 ± 0.070 ^b^
Passion fruit	0.29 ± 0.080 ^c^	1.37 ± 0.080 ^b^	0.20 ± 0.080 ^c^	2.53 ± 0.080 ^a^
Exotic fruits	0.43 ± 0.079 ^b^	2.56 ± 0.079 ^a^	0.41 ± 0.079 ^b^	0.47 ± 0.079 ^b^
Yellow fruits	0.23 ± 0.063 ^b^	1.45 ± 0.063 ^a^	0.28 ± 0.063 ^b^	0.28 ± 0.063 ^b^
Red fruits	0.15 ± 0.051 ^b^	2.20 ± 0.051 ^a^	0.13 ± 0.051 ^b^	0.14 ± 0.051 ^b^
Metallic	0.32 ± 0.063 ^b^	0.50 ± 0.063 ^b^	0.33 ± 0.063 ^b^	1.70 ± 0.063 ^b^
Taste				
Sour	4.89 ± 0.087 ^a^	3.48 ± 0.087 ^b^	3.17 ± 0.087 ^bc^	2.97 ± 0.065 ^c^
Bitter	1.20 ± 0.059 ^a^	1.70 ± 0.059 ^a^	0.47 ± 0.059 ^c^	1.82 ± 0.059 ^a^
Salty	1.74 ± 0.066 ^ab^	1.58 ± 0.066 ^b^	1.86 ± 0.066 ^a^	1.84 ± 0.066 ^a^
Sweet	4.12 ± 0.080 ^ab^	4.26 ± 0.080 ^a^	3.87 ± 0.80 ^bc^	3.80 ± 0.80 ^c^
Astringent	0.41 ± 0.073 ^b^	0.49 ± 0.073 ^b^	0.37 ± 0.073 ^b^	0.98 ± 0.073 ^a^

The estimated means in the same line identified by different letters are considered significantly different (Tukey test, *p* < 0.05).

**Table 3:** The mean intensity (±standard error) of each taste, astringent and metallic attribute in the “after tasting” segment for the nutritional supplements.

Attributes	CN1	CN2	CN3	CN4
Astringent	0.516 ± 0.065 ^b^	0.418 ± 0.065 ^b^	0.462 ± 0.065 ^b^	1.113 ± 0.065 ^a^
Metallic	0.261 ± 0.045 ^b^	0.282 ± 0.045 ^b^	0.179 ± 0.045 ^b^	1.671 ± 0.045 ^a^
Salty	1.500 ± 0.068 ^a^	1.018 ± 0.068 ^b^	0.953 ± 0.068 ^b^	1.112 ± 0.068 ^b^
Sour	2.005 ± 0.082 ^a^	1.766 ± 0.082 ^a^	1.992 ± 0.082 ^a^	1.857 ± 0.082 ^a^
Bitter	1.406 ± 0.072 ^ab^	1.647 ± 0.072 ^a^	0.369 ± 0.072 ^c^	1.274 ± 0.072 ^b^
Sweet	2.751 ± 0.078 ^b^	3.313 ± 0.078 ^a^	2.841 ± 0.78 ^b^	2.694 ± 0.78 ^b^

The estimated means in the same line identified by different letters are considered significantly different (Tukey test, *p* < 0.05).

**Table 4:** The mean intensity of each taste, astringent and metallic sensory attribute during and after the ingestion of the four nutritional supplements with and without a nose-clip.

	CN1			CN2			CN3			CN4		
Attributes	With nose-clip	Without nose-clip	*p*-value	With nose-clip	Without nose-clip	*p*-value	With nose-clip	Without nose-clip	*p*-value	With nose-clip	Without nose-clip	*p*-value
	During tasting											
Sour	5.03	4.91	0.168	3.68	3.49	0.258	3.28	3.15	0.309	3.45	2.96	0.001
Bitter	1.44	1.23	0.035	2.10	1.68	**<0.0001**	1.17	0.44	**<0.0001**	1.37	1.72	0.005
Astringent	0.97	0.44	**<0.0001**	0.79	0.49	0.064	0.48	0.37	0.396	1.39	0.96	**<0.0001**
Metallic	0.20	0.35	0.061	0.20	0.50	0.007	0.12	0.32	0.022	0.24	1.66	**<0.0001**
Salty	1.98	1.75	0.017	1.39	1.59	0.128	1.79	1.83	0.744	1.90	1.83	0.617
Sweet	3.61	4.13	**<0.0001**	3.52	4.25	**<0.0001**	3.35	3.83	**<0.0001**	3.62	3.78	0.266
	After tasting											
Sour	3.68	1.99	**<0.0001**	1.46	1.77	0.040	1.34	2.02	**<0.0001**	1.89	1.82	0.63
Bitter	1.29	1.42	0.093	2.77	1.57	**<0.0001**	0.69	0.37	0.004	0.97	1.21	0.06
Astringent	1.27	0.56	**<0.0001**	0.53	0.43	0.503	0.62	0.46	0.149	1.44	1.04	0.009
Metallic	0.18	0.28	0.180	0.25	0.28	0.718	0.19	0.20	0.852	0.16	1.60	**<0.0001**
Salty	1.14	1.46	0.015	0.93	0.97	0.791	0.84	0.95	0.400	0.85	1.14	0.061
Sweet	1.96	2.69	**<0.0001**	2.19	3.28	**<0.0001**	2.16	2.83	**<0.0001**	2.28	2.62	0.005

ANOVA and Tukey test; significant *p*-values are in bold (*p* < 0.05).

**Table 5:** Main chemical composition of nutritional supplements.

	CN1	CN2	CN3	CN4
Vitamins				
B1 (Thiamine phosphate; Thiamine hydrochloride)	15 mg	1.4 mg		3.1 mg
B2 (Riboflavin phosphate)	15 mg	1.6 mg		4 mg
B3 (Nicotinamide)	50 mg	18 mg		45 mg
B5 (Calcium pantothenate)	23 mg	6 mg		17 mg
B6 (Pyridoxine hydrochloride)	10 mg	2 mg		4 mg
B8 (D-biotin)	0.15 mg	0.15 mg		145 µg
B9 (Folic acid)	0.4 mg	200 µg		430 µg
B12 (Cobalamin (0.1%))	10 µg	1 µg		3 µg
C (Ascorbic acid)	500 mg	60 mg	1000 mg	180 mg
A (Palmitate (100%))				0.86 mg
D (Cholecalciferol (100%))				12 mg
E (Tocopherol acetate (50%))				5 µg
K (Phylloquinone (5%))				20 µg
Minerals				
Calcium (Calcium carbonate; Calcium phosphate)	100 mg	100 mg		120 mg
Magnesium (Magnesium carbonate; Magnesium sulphate; Magnesium oxide)	100 mg	100 mg		80 mg
Iron (Iron pyrophosphate)				8 mg
Iodide				150 µg
Zinc (Zinc citrate trihydrate)	10 mg	5 mg		4.4 mg
Manganese (Manganese sulphate)				500 µg
Copper (Copper citrate hemipentahydrate)				1 mg
Molybdenum (Sodium molybdenum)				50 µg
Selenium (Sodium selenite)				55 µg
Caffeine		40 mg		

**Table 6:** Sensory attributes along with their definitions and their reference samples used in the sensory study (olfactory and gustatory modalities).

Consumption Phases and Assessment Guidelines	Sensory Modalities Assessed	Descriptors	Definitions	Sensory References
Before tasting «Place the tablet in the provided cup. Wait for the tablet to completely dissolve in water, indicated by the visual disappearance of the tablet in the cup. Bring the sample to your nose and record the olfactory intensity of the specified attributes.»	Olfactory (9)	Fresh orange	Characteristic odour of freshly squeezed orange	NI
		Mature orange	Characteristic odour of an orange in an advanced state of decomposition (presence of mould)	NI
		Chemical orange	Characteristic odour of certain confectionery products or carbonated orange-flavoured drinks.	NI
		Mandarin	Characteristic odour of the fruit and/or its juice	NI
		Passion fruit	Characteristic odour of the fruit and/or its juice	NI
		Lemon	Characteristic odour of the fruit and/or its juice	NI
		Exotic fruits	Characteristic odour of a blend of different exotic fruits (mango, pineapple, banana) squeezed together	NI
		Red fruits	Characteristic odour of a blend of different pressed red fruits (strawberry, raspberry)	NI
		Yellow fruits	Characteristic odour of a blend of different pressed yellow fruits (peach, apricot, nectarine)	NI
During tasting «After evaluating the olfactory descriptors, proceed with the assessment of the gustatory descriptors. To do so, bring the sample to your mouth and promptly record the gustatory intensity of the specified attributes.»	Gustatory (5)	Sour	Elemental flavour produced by dilute aqueous solutions of various substances such as citric acid or tartaric acid	Negative terminal (0): Evian water Positive terminal (10): Citric acid (1.8 g/L) + Evian water
		Sweet	A basic flavour produced by dilute solutions of various substances such as sucrose or aspartame	Negative terminal (0): Evian water Positive terminal (10): Sucrose (80 g/L) + Evian water
		Salty	Elemental flavour caused by diluted substances such as sodium chloride	Negative terminal (0): Evian water Positive terminal (10): Sodium chloride (6 g/L) + Evian water
		Bitter	Elemental flavour caused by dilute solutions of various substances such as caffeine or quinine hydrochloride	Negative terminal (0): Evian water Positive terminal (10): Caffeine (1.5 g/L) + Evian water
		Astringent	A complex sensation, accompanied by a contraction, stretching or wrinkling of the oral mucosa, produced by substances such as the tannins in wine	Negative terminal (0): Evian water Positive terminal (10): Tannic acid (1 g/L) + Evian water

NI: not included.

**Table 7:** Sensory attributes along with their definitions and their reference samples used in the sensory study (aromatic and aftertaste modalities).

Consumption Phases and Assessment Guidelines	Sensory Modalities Assessed	Descriptors	Definitions	Sensory References
During tasting «After evaluating the olfactory descriptors, proceed with the assessment of the aromatic descriptors. To do so, bring the sample to your mouth and promptly record the aromatic intensity of the specified attributes.»	Aromatic (10)	Fresh orange	Characteristic aroma of freshly squeezed orange	NI
		Mature orange	Characteristic of an orange in an advanced state of decomposition (presence of mould)	NI
		Chemical orange	Characteristic aroma of certain confectionery products or carbonated orange-flavoured drinks	NI
		Mandarin	Characteristic aroma of the fruit and/or its juice	NI
		Passion fruit	Characteristic aroma of the fruit and/or its juice	NI
		Lemon	Characteristic aroma of the fruit and/or its juice	NI
		Exotic fruits	Characteristic aroma of a blend of different exotic fruits (mango, pineapple, banana) squeezed together	NI
		Red fruits	Characteristic aroma of a blend of different pressed red fruits (strawberry, raspberry)	NI
		Yellow fruits	Characteristic aroma of a blend of different pressed yellow fruits (peach, apricot, nectarine)	NI
		Metallic	Sensation caused by substances such as ferrous sulphate, similar to that felt when the tongue or oral mucosa is cut	Negative terminal (0): Evian water Positive terminal (10): Ferrous sulphate (0.1 g/L) + Evian water
After tasting «You will proceed with the evaluation of the persistence of gustatory descriptors. To do so, bring the sample to your mouth and record the gustatory intensity of the specified attributes 10 s after complete ingestion of the sample.»	Aftertaste (5)	Sour	Elemental flavour produced by dilute aqueous solutions of various substances such as citric acid or tartaric acid	Negative terminal (0): Evian water Positive terminal (10): Citric acid (1.8 g/L) + Evian water
		Sweet	A basic flavour produced by dilute solutions of various substances such as sucrose or aspartame	Negative terminal (0): Evian water Positive terminal (10): Sucrose (80 g/L) + Evian water
		Salty	Elemental flavour caused by diluted substances such as sodium chloride	Negative terminal (0): Evian water Positive terminal (10): Sodium chloride (6 g/L) + Evian water
		Bitter	Elemental flavour caused by dilute solutions of various substances such as caffeine or quinine hydrochloride	Negative terminal (0): Evian water Positive terminal (10): Caffeine (1.5 g/L) + Evian water
		Astringent	A complex sensation, accompanied by a contraction, stretching or wrinkling of the oral mucosa, produced by substances such as the tannins in wine	Negative terminal (0): Evian water Positive terminal (10): Tannic acid (1 g/L) + Evian water

NI: not included.

## Data Availability

Data are contained within the article and Supplementary Materials.

## Supplementary Materials

The following supporting information can be downloaded at: https://www.mdpi.com/article/10.3390/molecules30040854/s1, Table S1. ANOVA results for the evaluation without nose clip (subject + product + subject × product + error) to identify which descriptors significantly differentiate the products (orthonal odour, retronasal odour, taste and aftertaste) “before, during and after tasting”. Table S2. ANOVA results for the evaluation with nose clip (subject + product + subject × product + error) to identify which descriptors significantly differentiate the products (taste, astringent and metallic sensory attribute) “during and after tasting”.
