# Supplementary material for: Sensory Evaluation of Effervescent Nutritional Supplements: Identification and Characterisation of Off-Tastes
Source: Molecules. 2025 Feb 13;30(4):854. doi: 10.3390/molecules30040854 (PMC11858554; doi:10.3390/molecules30040854)
Supplement: Supplementary file 1 [file molecules-30-00854-s001.zip › molecules-3428177-supplementary.pdf]

Table S1. ANOVA results for the evaluation without nose clip (subject + product + subject*product + error) to identify which descriptors significantly differentiate the products (orthonal odour, retronasal odour, taste and aftertaste) "before, during and after tasting".

Attribut	Subject		Product		Subject*Product	
	F	*p*-Value	F	*p*-Value	F	*p*-Value
**Orthonasal odour**						
Chemical orange	0.51	0.944	220.18	**< 0,0001**	3.22	**< 0,0001**
Fresh orange	0.84	0.640	100.26	**< 0,0001**	3.38	**< 0,0001**
Mature orange	0.88	0.607	95.83	**< 0,0001**	2.28	**< 0,0001**
Lemon	6.27	**< 0,0001**	2.27	0.090	0.84	0.755
Mandarine	2.02	**0.024**	29.77	**< 0,0001**	1.67	**0.019**
Passion fruit	1.04	0.431	199.58	**< 0,0001**	4.35	**< 0,0001**
Exotic Fruits	1.27	0.242	96.90	**< 0,0001**	2.61	**< 0,0001**
Yellow fruit	3.31	**< 0,0001**	82.15	**< 0,0001**	0.33	1
Red fruit	1.88	**0.039**	256.68	**< 0,0001**	2.89	**< 0,0001**
**Retronasal odour**						
Chemical orange	1.32	0.211	299.44	**< 0,0001**	1.59	**0.03**
Fresh orange	1.73	0.062	159.24	**< 0,0001**	2.45	**0.001**
Mature orange	1.32	0.209	87.93	**< 0,0001**	6.85	**< 0,0001**
Lemon	5.88	**< 0,0001**	4.86	**0.005**	0.78	0.831
Mandarine	2.88	**0.001**	30.85	**< 0,0001**	0.93	0.610
Passion fruit	1.19	0.304	99.33	**< 0,0001**	1.87	**0.006**
Exotic Fruits	1.55	0.108	100.83	**< 0,0001**	1.80	**0.009**
Yellow fruit	1.62	0.086	41.94	**< 0,0001**	2.11	**0.001**
Red fruit	1.84	**0.043**	191.20	**< 0,0001**	2.13	**0.001**
Metallic	1.97	**0.028**	52.24	**< 0,0001**	2.09	**0.002**
**Taste**						
Sour	3.2576	**0.001**	67.75	**< 0,0001**	1.47	0.061
Bitter	2.24	**0.012**	38.29	**< 0,0001**	2.85	**< 0,0001**
Salty	2.28	**0.01**	1.26	0.297	2.96	**< 0,0001**
Sweet	1.62	0.088	4.61	**0.006**	1.56	**0.037**
Astringent	4.46	**< 0,0001**	11.74	**< 0,0001**	1.26	0.176
**Aftertaste**						
Sour	2.53	**0.004**	1.11	0.352	1.74	**0.013**
Bitter	0.75	0.741	24.19	**< 0,0001**	2.48	**0.001**
Salty	7.29	**< 0,0001**	7.10	**0.0004**	1.84	**0.007**
Sweet	1.98	**0.028**	6.78	**0.001**	1.90	**0.005**
Astringent	2.58	**0.004**	10.30	**< 0,0001**	2.56	**< 0,0001**
Metallic	2.22	**0.012**	63.98	**< 0,0001**	3.93	**< 0,0001**

Significant p-values are in bold ($p < 0.05$).

Table S2. ANOVA results for the evaluation with nose clip (subject + product + subject*product + error) to identify which descriptors significantly differentiate the products (taste, astringent and metallic sensory attribute) "during and after tasting".

Attribut	Subject		Product		Subject*Product	
	**F**	***p*-Value**	**F**	***p*-Value**	**F**	***p*-Value**
**Taste**						
Sour	1.34	0.213	121.41	**< 0,0001**	1.70	**0.022**
Bitter	1.98	**0.035**	60.74	**< 0,0001**	1.84	**0.010**
Salty	1.63	0.097	18.97	**< 0,0001**	0.72	0.885
Sweet	1.67	0.086	1.54	0.216	2.15	**0.002**
Astringent	1.58	0.111	17.09	**< 0,0001**	2.51	**0.001**
Metallic	3.17	**0.001**	1.94	0.135	1.18	0.263
**Aftertaste**						
Sour	0.84	0.640	172.89	**< 0,0001**	2.47	**0.001**
Bitter	1.23	0.279	224.05	**< 0,0001**	1.63	**0.031**
Salty	3.11	**0.001**	4.31	**0.009**	1.17	0.276
Sweet	0.94	**0.532**	4.69	**0.006**	1.21	0.228
Astringent	2.78	**0.003**	30.35	**< 0,0001**	1.95	**0.006**
Metallic	6.42	**< 0,0001**	1.29	0.287	0.63	0.953

Significant p-values are in bold ($p < 0.05$).
